# Supplementary material for: Siderophore-inspired chelator hijacks uranium from aqueous medium
Source: Nat Commun. 2019 Feb 18;10:819. doi: 10.1038/s41467-019-08758-1 (PMC6379418; doi:10.1038/s41467-019-08758-1)
Supplement: Supplementary file 1 — Supplementary Information [file 41467_2019_8758_MOESM1_ESM.pdf]

Supplementary Information

Siderophore-inspired chelator hijacks uranium from aqueous medium

Ivanov et al.

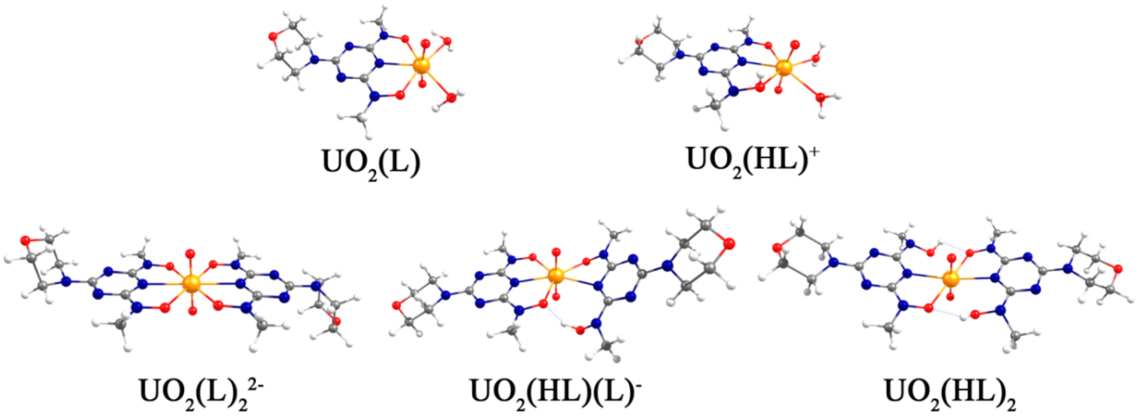

Supplementary Figure 1. Structures of the most stable uranyl complexes with 1,3,5-triazine hydroxylamine (H_2BHT) obtained after geometry optimization at the M06/SSC/6-311++G** level of theory.

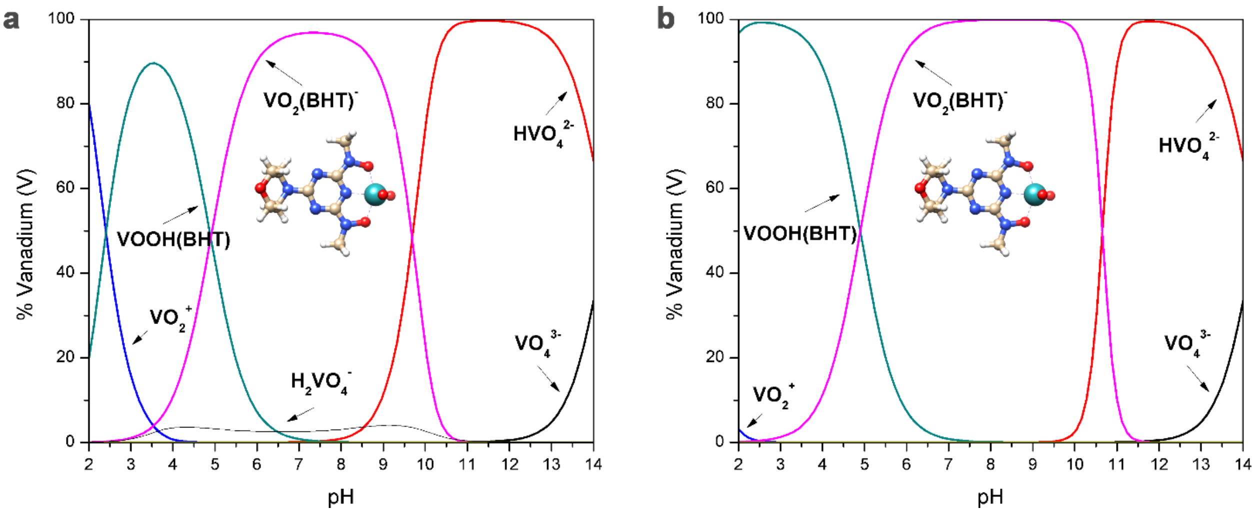

Supplementary Figure 2. Species distribution diagrams generated using theoretically predicted log β^{theor} values of vanadium complexes (Supplementary Table 5) for **a** 1 mM [H_2BHT] and 1 mM [V] ([H_2BHT]/[V]= 1/1 ratio) and **b** 100 mM [H_2BHT] and 1 mM [V] ([H_2BHT]/[V]= 100/1 ratio) as a function of pH. The most stable species at pH 5-10 (DFT optimized $VO_2(BHT)^-$ complex) is shown here. Color scheme: V(V), turquoise; O, red; N, navy blue; C, beige; H, white.

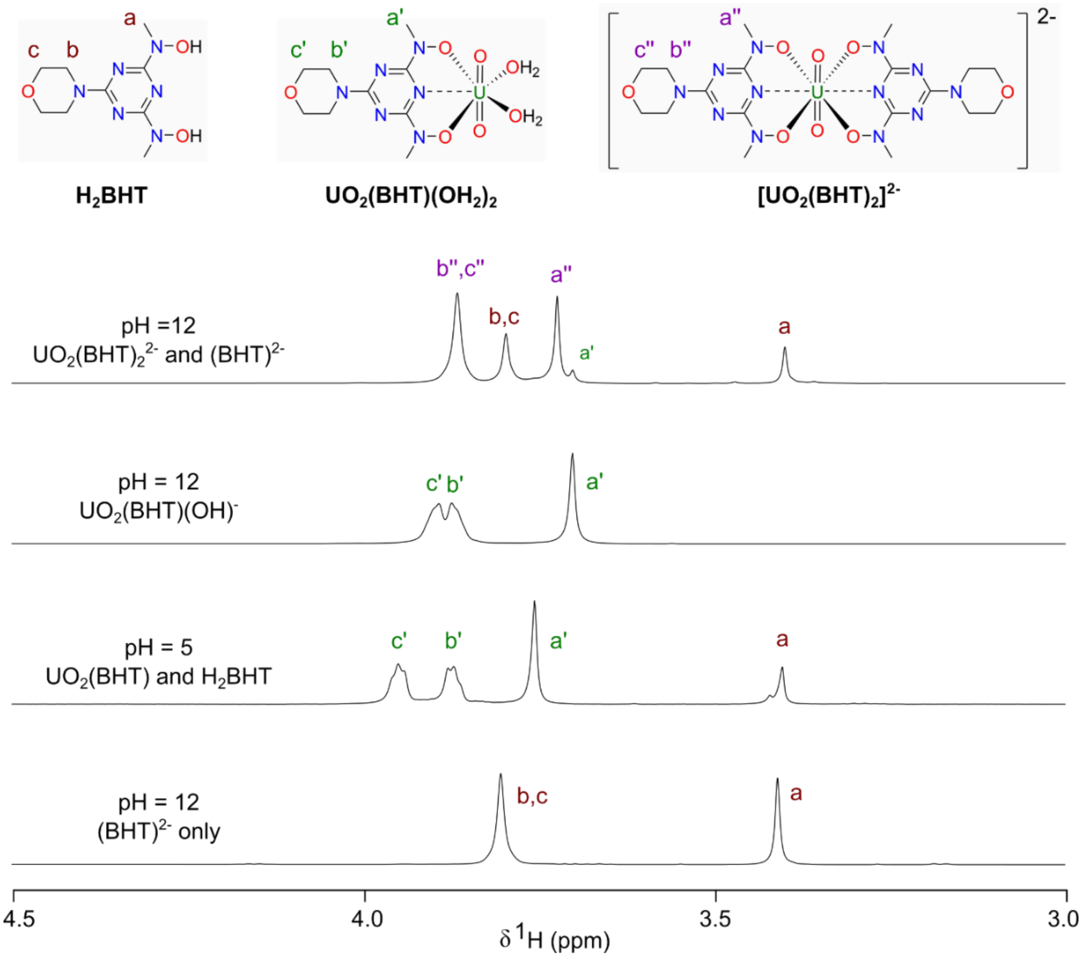

Supplementary Figure 3. ^{1}H NMR spectra of the H_2BHT complexation with uranyl in aqueous solution.

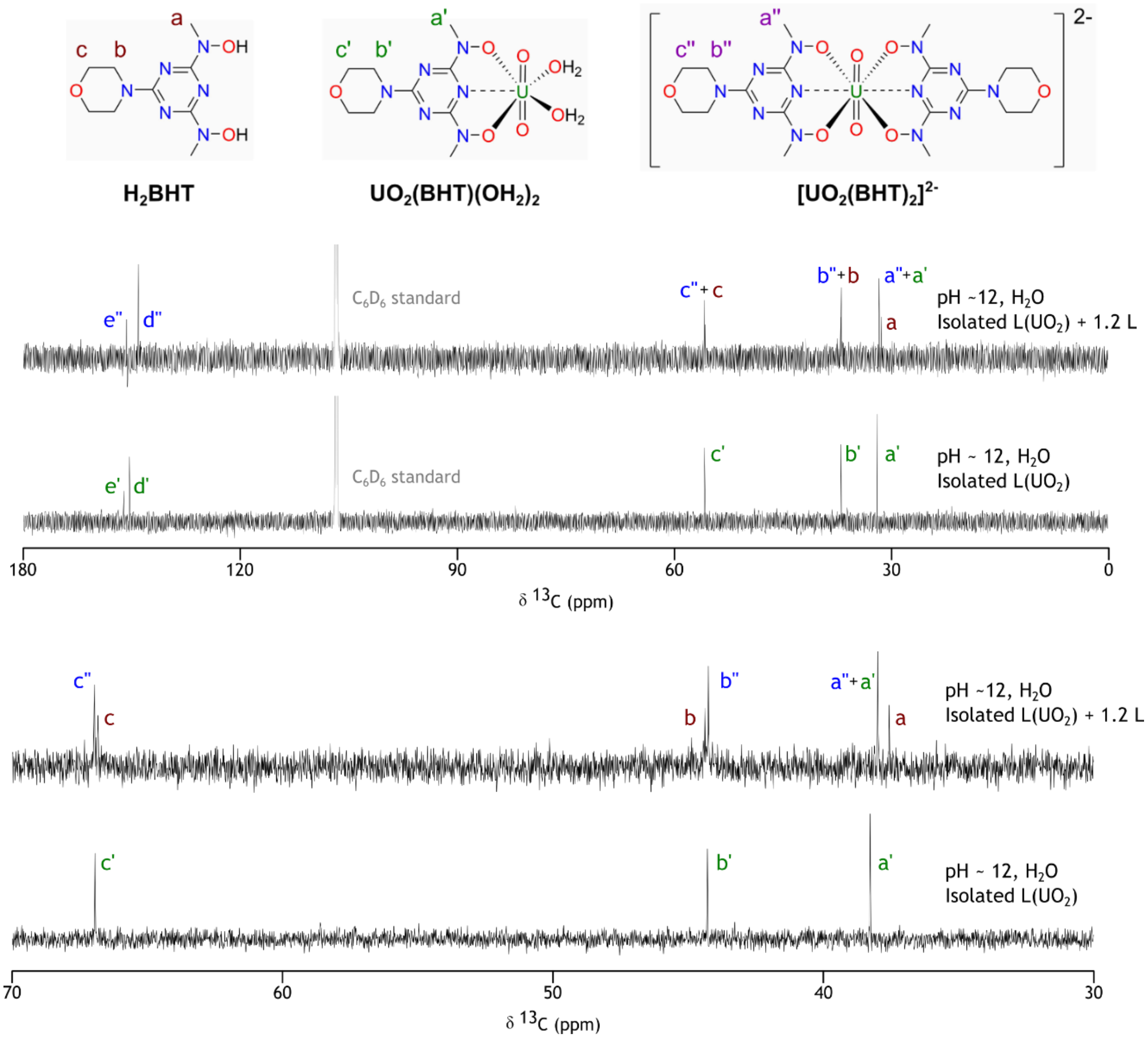

Supplementary Figure 4. ^{13}C NMR spectra of H_2BHT/UO_2^{2+} in water (upper: full spectra; lower: aliphatic region only).

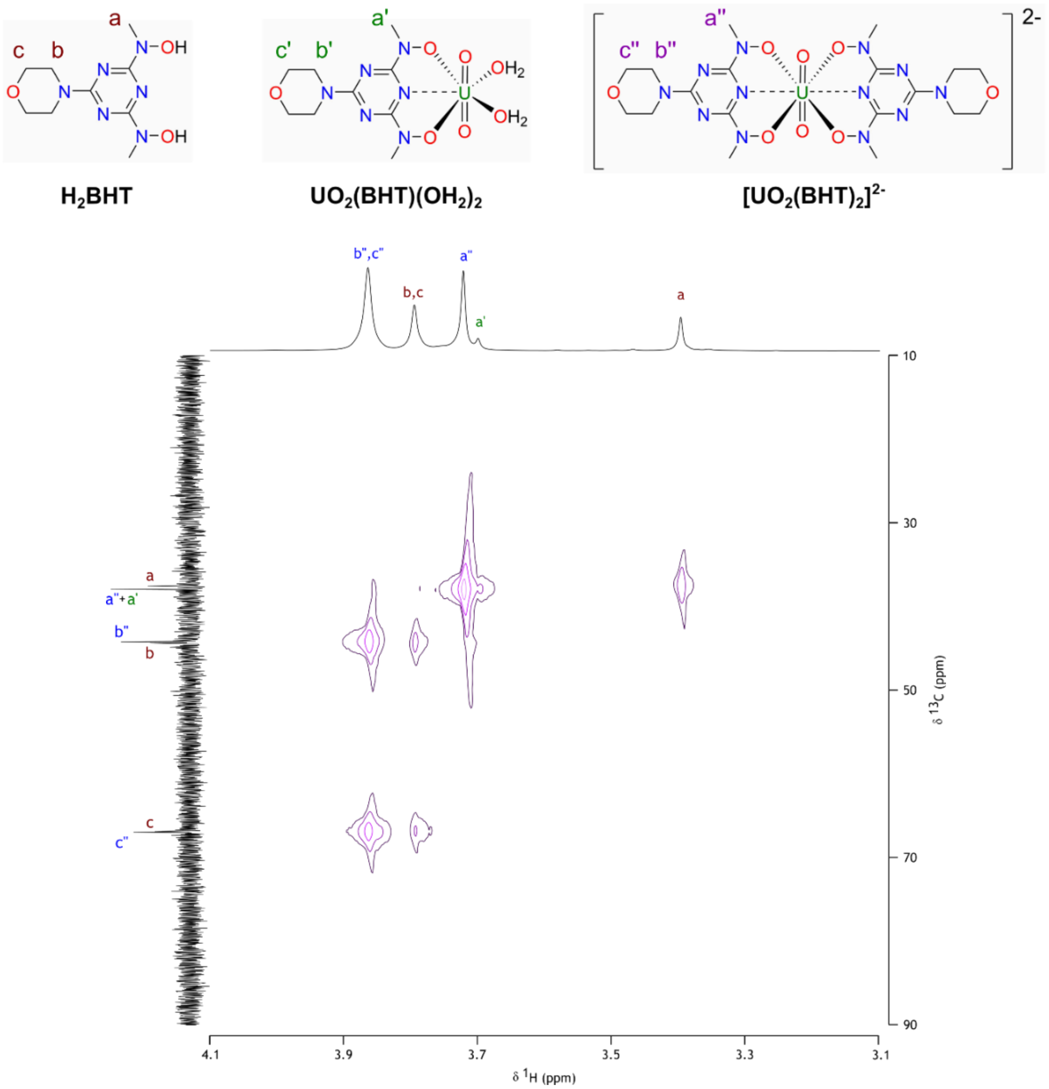

Supplementary Figure 5. ^{1}H-^{13}C HSQC spectrum of the aliphatic region, confirming peak assignments.

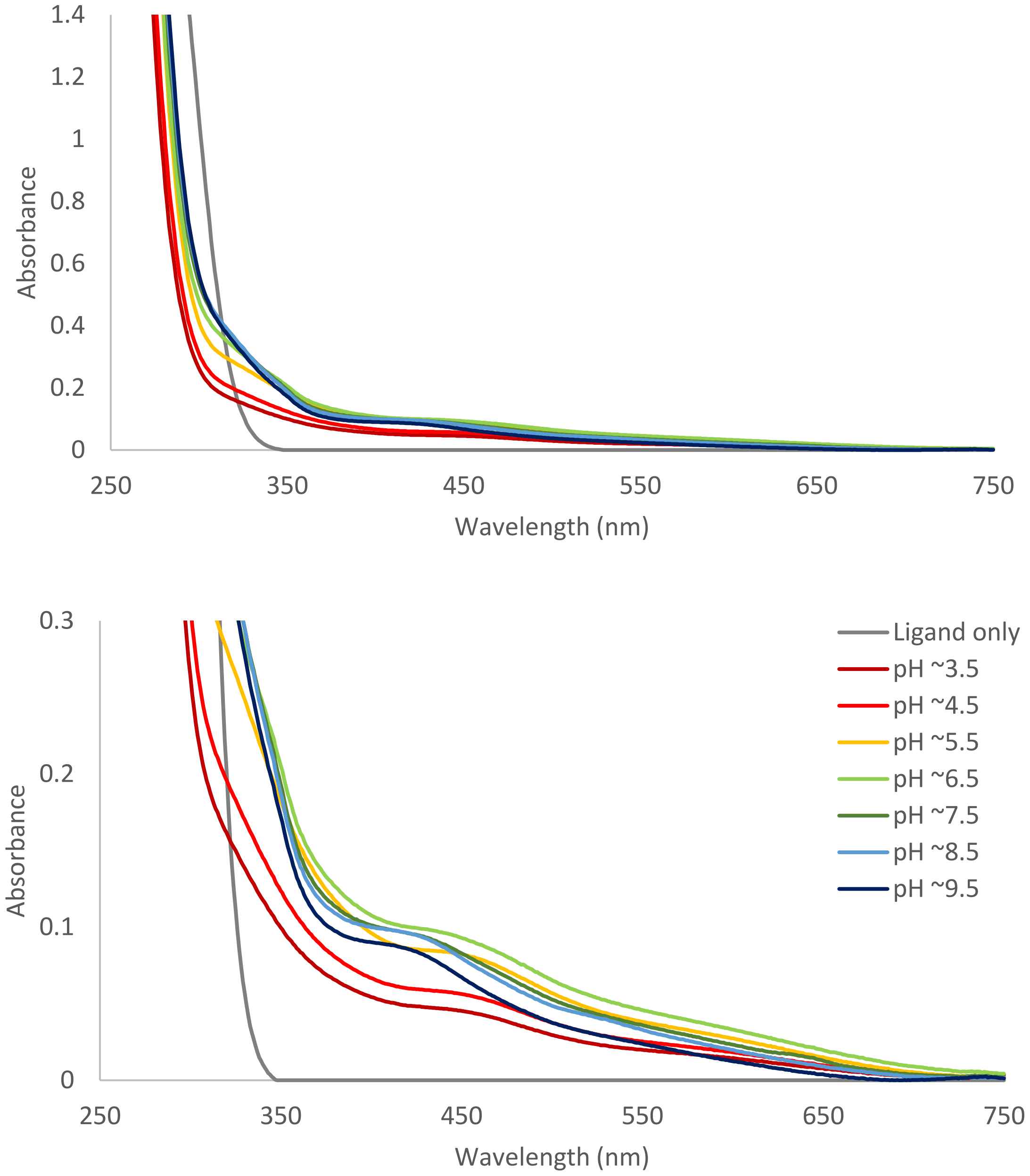

Supplementary Figure 6. UV-Visible absorption spectra of uranyl – H_2BHT solutions. The lower spectrum is an inset of the upper spectrum. Conditions: $[H_2BHT] = 0.8$ mM, $[U(VI)] = 0.4$ mM (except ligand only solution), 2 mm cuvette.

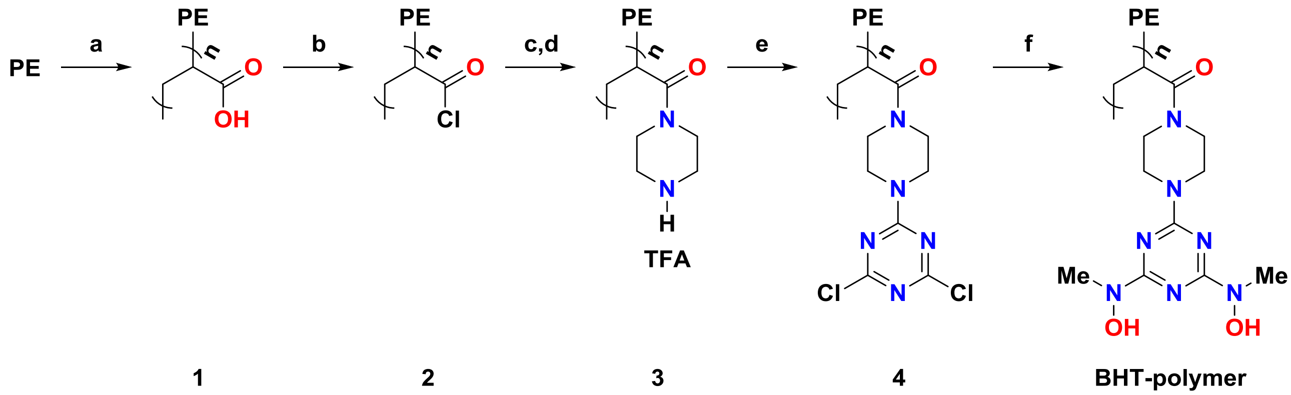

Supplementary Figure 7. Synthesis of H_2BHT polymeric adsorbent. Conditions: a) electron beam/acrylic acid DMSO, -78 °C to RT b) $SOCl_2$, CH_2Cl_2, 40 °C, 24 h c) N-BOC-piperazine, Hünig's base, CH_2Cl_2, 0 °C to RT, 12 h d) trifluoroacetic acid, CH_2Cl_2, RT, 2 h e) 1,3,5 trichlorotriazene, N,N-diisopropylethylamine base, acetone, -40 °C to 0 °C, 24 h f) MeNHOH*HCl, N,N-diisopropylethylamine , THF 0 °C to RT, 24 h.

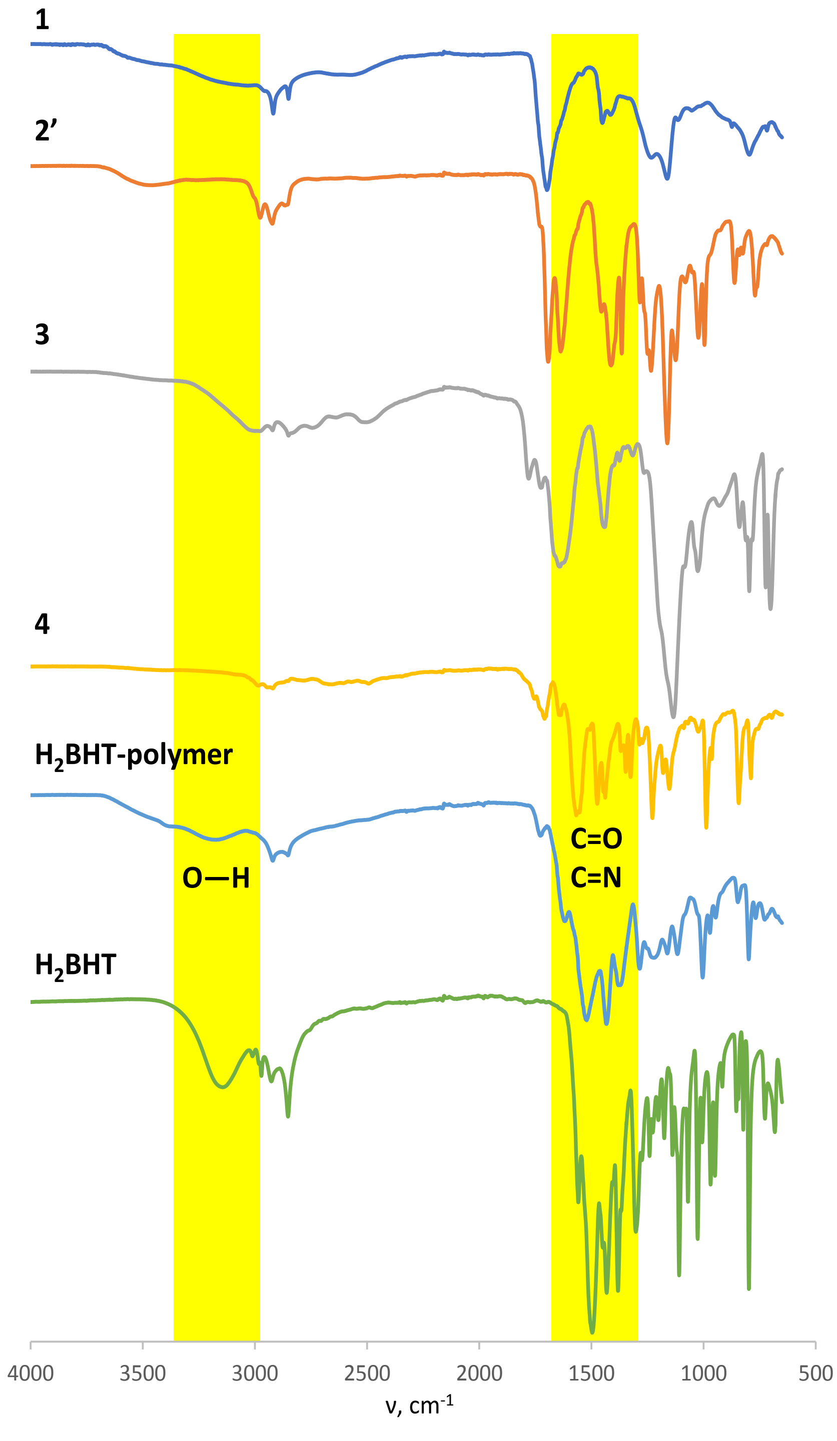

Supplementary Figure 8. Comparative IR spectra of intermediates **1**–**4**, H_2BHT-polymer and H_2BHT small molecule.

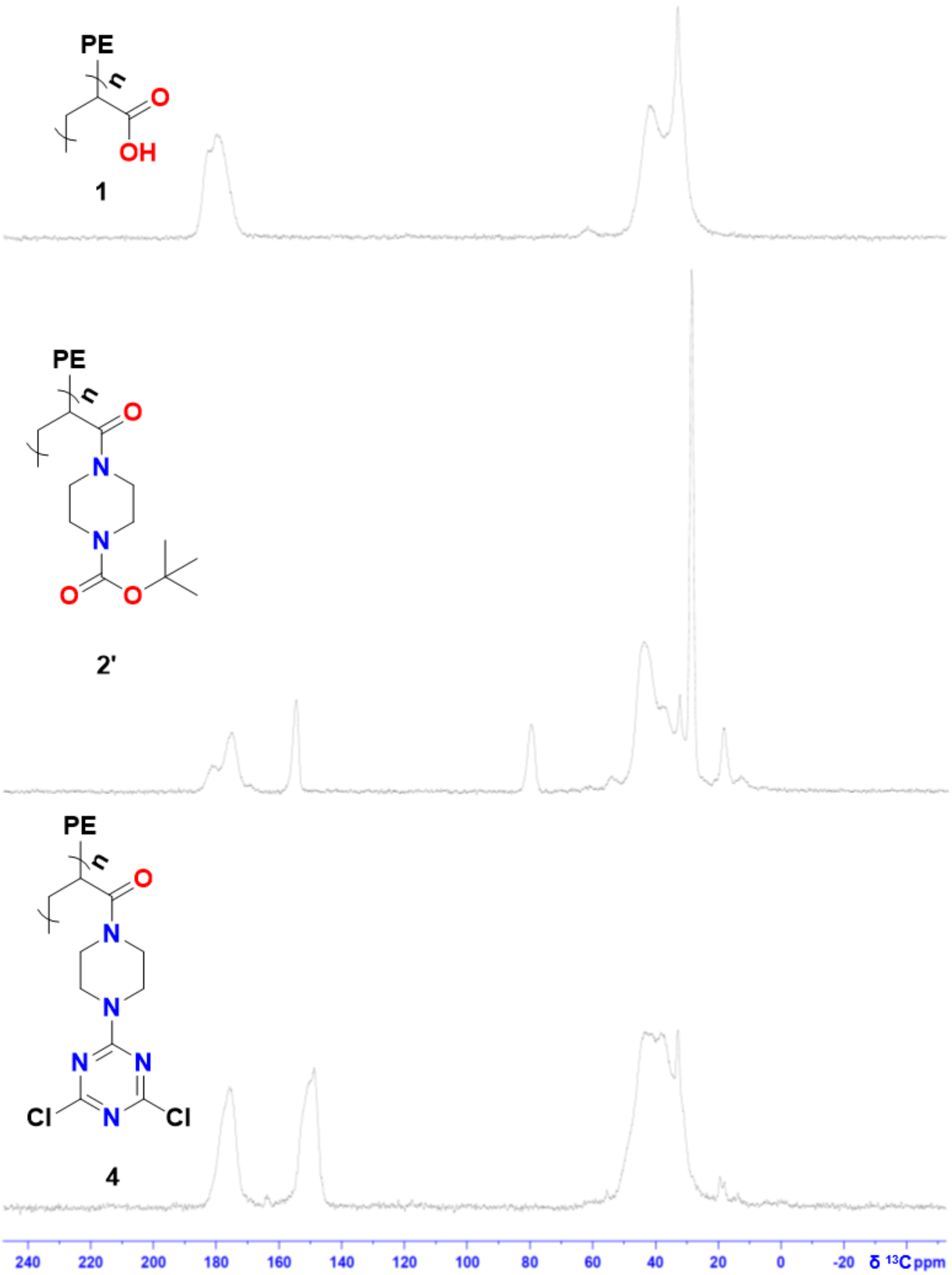

Supplementary Figure 9. δ ^{13}C CP/MAS solid-state NMR spectra of intermediates **1**, **2’** and **4**.

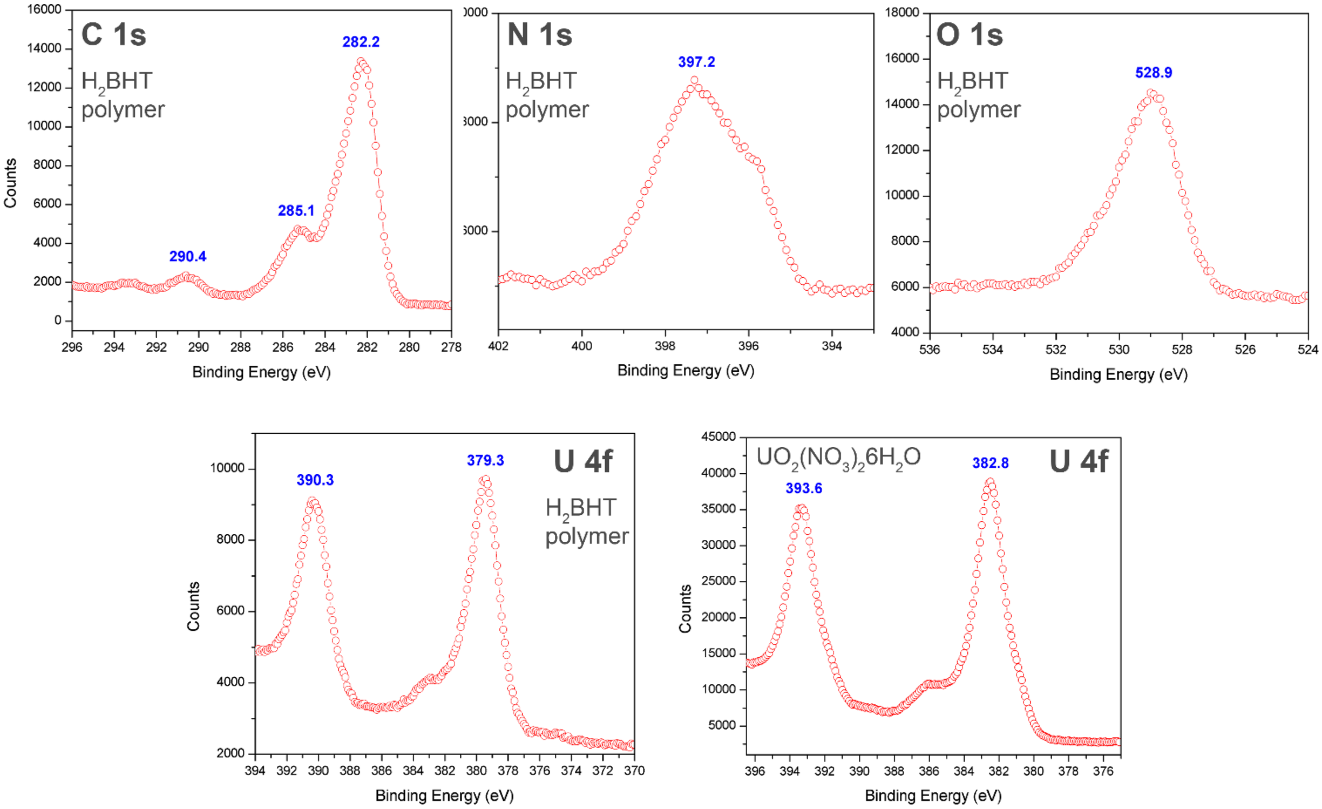

Supplementary Figure 10. C1s, N1s, O1s, and U4f XPS spectra of the uranium-contacted H_2BHT-polymer. The U4f XPS spectrum of $UO_2(NO_3)_2 \cdot 6H_2O$ is given for comparison.

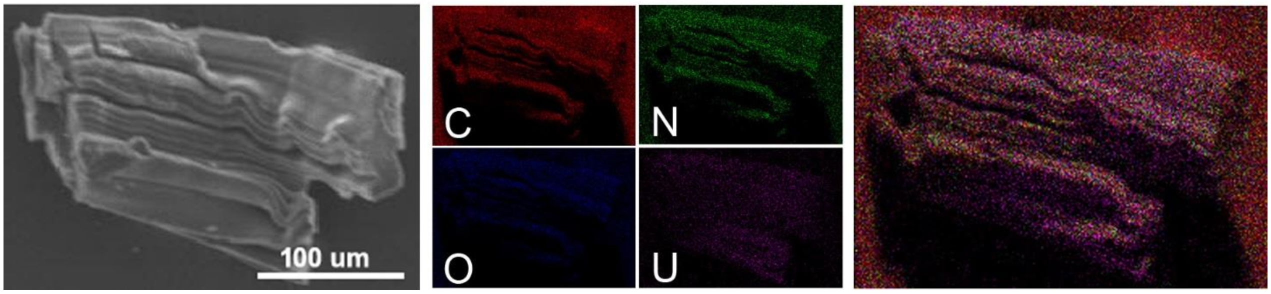

Supplementary Figure 11. SEM image of the uranium-contacted H_2BHT-polymer and corresponding EDX elemental mapping images of C (red), N (green), O (blue), U (purple), and the elemental overlap.

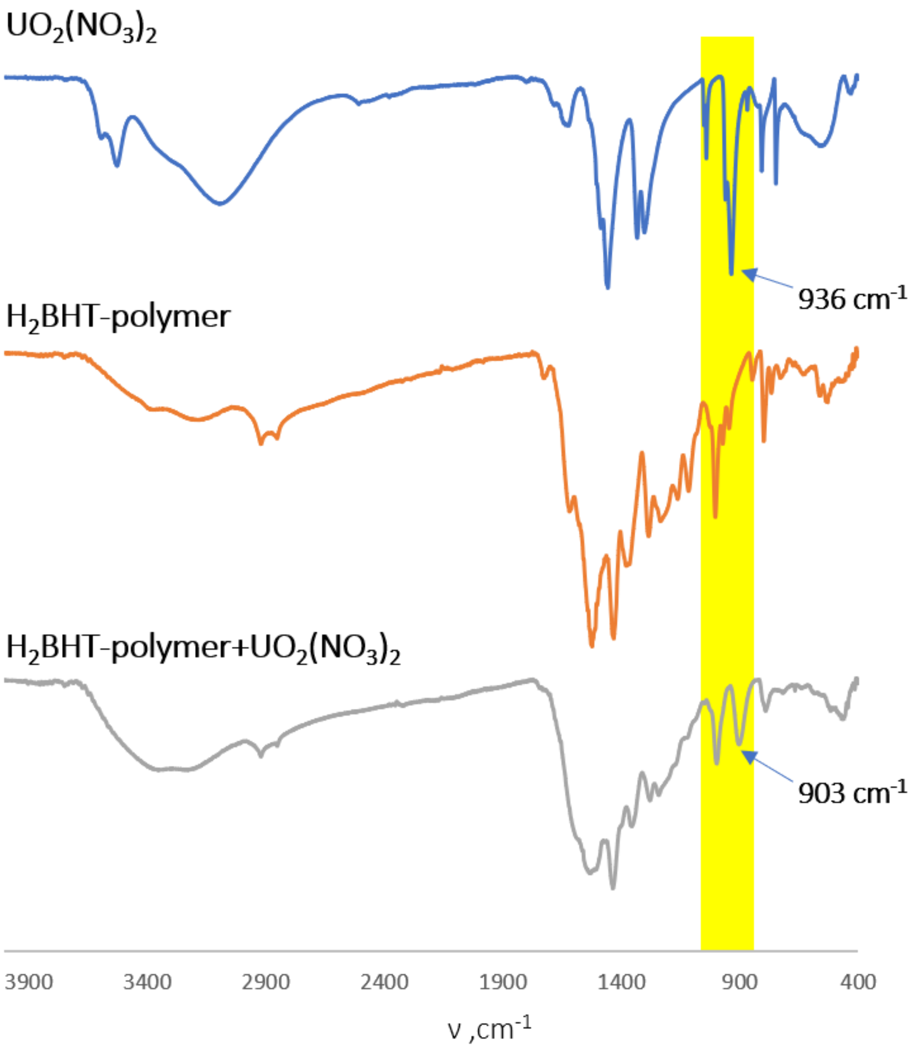

Supplementary Figure 12. Comparative IR spectra of $UO_2(NO_3)_2$, H_2BHT-polymer and H_2BHT-polymer contacted with $UO_2(NO_3)_2$ solution. Note $[O=U=O]^{2+}$ moiety antisymmetric stretching mode frequency shifts from 936 cm^{-1} to 903 cm^{-1} upon complexation with H_2BHT-functionalized polymer.

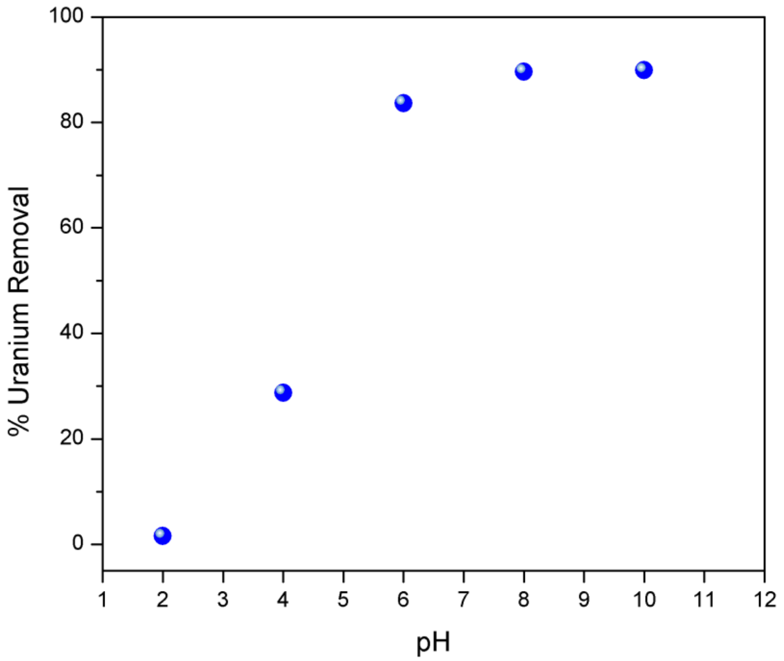

Supplementary Figure 13. The pH dependence of H_2BHT adsorbent (5 mg) uptake capacities (expressed in % U removal) with starting uranium concentration of 10 ppm (100 mL) and 24 h contact time. The pH values were adjusted with NaOH and/or HNO_3. As can be seen, the highest adsorption is achieved at pH 6-10. The pH 6 was chosen for the sorption and selectivity studies to avoid possible issues with the precipitation of uranium/vanadium complexes at higher uranium/vanadium concentrations for the pH range 8-10.

Supplementary Table 1. Crystallographic data for $UO_2(BHT)\cdot 2H_2O$.

Formula	$C_9H_{20}N_6O_8U$
Formula weight (g·mol^{-1})	578.32
Space group	Monoclinic, $P2_1/c$
a (Å)	11.476(3)
b (Å)	13.364(3)
c (Å)	11.110(3)
α (°)	90
β (°)	106.465(4)
γ (°)	90
V (Å^3)	1634.1(7)
Z	4
ρ_{calc} (g·cm^{-3})	2.351
μ (mm^{-1})	9.986
F(000)	1088
Crystal size (mm × mm × mm)	0.100 × 0.060 × 0.060
Crystal color and form	Dark brown block
Theta range for data collection	1.850 to 25.371°
Index ranges	-13<=h<=13, -16<=k<=16, -13<=l<=13
Reflections collected	44023
Independent reflections	2983 [R(int) = 0.0386]
R1 ($I > 2\sigma(I)$)	0.0138
wR2 ($I > 2\sigma(I)$)	0.0345
R1 (all data)	0.0139
wR2 (all data)	0.0345
GoF	1.175
Largest difference peak/hole (e·Å^3)	0.619 / -0.891

Supplementary Table 2. Selected bond lengths and angles in the $UO_2(BHT)\cdot 2H_2O$ crystal structure.

Bond length (Å) or angle (°)	$UO_2(BHT)\cdot 2H_2O$
M–O (ligand)	2.362(2), 2.410(2)
M–N	2.439(2)
M=O (oxo)	1.787(2), 1.779(2)
M–O (water)	2.368(2), 2.375(2)
∠O–M–O (ligand)	126.61(7)
∠O=M=O (oxo)	175.70(9)

Supplementary Table 3. Comparison of the strengths of ligand-uranyl interactions in complexes using natural bond orbital (NBO) method. Second-order stabilization energies ($E^{(2)}$, kcal mol^{-1}) indicate comparable uranyl binding affinities of the triazine hydroxylamine (H_2BHT) and imide-dioxime (H_3IDO) ligands.

Complex	Donor NBO → Acceptor NBO in UO_2^{2+} complexes (kcal mol^{-1})[1]				Charge on UO_2^{2+} unit
	**LP$_N$→n*$_U$ (central nitrogen)**	**LP$_O$→n*$_U$ (oxygens oxime groups)**	**LP$_O$→n*$_U$ (water)**	**Total**	
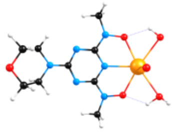 $UO_2(BHT)(H_2O)_2$	94.3	259.1	104.5	457.9	+0.81
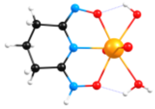 $UO_2(HIDO)(H_2O)_2$	90.7	282.9	100.8	474.4	+0.80

[1]The unstarred and starred labels correspond to Lewis (donor) and non-Lewis (acceptor) NBOs, respectively. Functional groups of the ligand contributing to the particular interaction are shown in parentheses. LP denotes an occupied lone pair; n*$_U$ denotes vacant U orbitals.

Supplementary Table 4. Protonation constants for glutaroimide-dioxime (H_3IDO) and bis-(hydroxylamino)-1,3,5-triazine (H_2BHT).

	pK_a (25 °C, 0.5 M NaCl)	
	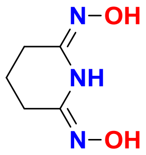	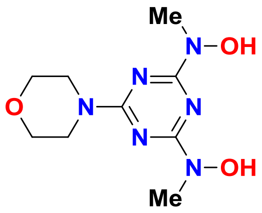
$H^+ + L^{2-} = HL^-$	12.06 ± 0.23	10.40 ± 0.14
$2H^+ + L^{2-} = H_2L$	22.76 ± 0.31	18.38 ± 0.23
$3H^+ + L^{2-} = H_3L^+$	24.88 ± 0.35	23.71 ± 0.28

Supplementary Table 5. Equilibrium constants for the aqueous vanadium (V) - H_2BHT reactions considered in the simulated speciation diagrams in Supplementary Fig.2.

Reactions	*log β*
$VO_4^{3-} + H^+ \leftrightarrows HVO_4^{2-}$	14.3[1]
$VO_4^{3-} + 2H^+ \leftrightarrows H_2VO_4^-$	22.85[1]
$VO_4^{3-} + 3H^+ \leftrightarrows H_3VO_4$	25.45[1]
$VO_4^{3-} + 4H^+ \leftrightarrows VO_2^+ + 2H_2O$	30.15[1]
$VO_4^{3-} + 4H^+ + BHT^{2-} \leftrightarrows VO_2(BHT)^- + 2H_2O$	47.45[2]
$VO_4^{3-} + 5H^+ + BHT^{2-} \leftrightarrows VOOH(BHT) + 2H_2O$	52.35[2]
$VO_4^{3-} + 8H^+ + 2BHT^{2-} \leftrightarrows V(BHT)_2^+ + 4H_2O$	72.05[2]

[1]The equilibrium constants at 25 °C and $I = 0$ are taken from "Pettersson, L. & Elvingson, K. in Vanadium Compounds, 711, 30–50, (1998)"; [2]Computationally predicted formation constants (see Supplementary Note 1). Experimental protonation constants for bis-(hydroxylamino)-1,3,5-triazine (H_2BHT) were taken from Supplementary Table 4.

Supplementary Table 6. Data used to plot the uranium sorption isotherm in Fig. 3d of the main text.

Starting Concentration (ppm)	Remaining Concentration (ppm)	Uptake Capacity (mg U/g adsorbent)
1.028	0.161	1.733
5.138	0.180	9.915
20.550	0.163	40.774
41.410	5.376	72.068
82.200	35.950	92.500
256.875	204.600	104.550

Supplementary Table 7. Competitive adsorption test (U(VI) vs. V(V) equal concentrations; 10 ppm; 200 mL) and regeneration with the uranium uptake capacities in uranyl solution (10 ppm, 400 mL).

	Remaining Uranium Concentration	Uranium Uptake Capacity	Percent Removal
U vs. V Test 1	0.153 ppm	39.388 mg g^{-1}	98.47%
U vs. V Test 2	0.154 ppm	39.384 mg g^{-1}	98.46%
Regeneration (3rd recycling)	0.093 ppm	79.256 mg g^{-1}	99.07%

*Change in the vanadium (V) concentration was not observed

Supplementary Table 8. Vanadium adsorption test (U(VI), 0.42×10^{-4} M vs. V(V), 1.15×10^{-4} M; 400 mL; [V]/[U] ~ 3/1 ratio).

	Starting Vanadium Concentration	Remaining Vanadium Concentration	Vanadium Uptake Capacity	Percent removal
V Test	1.15×10^{-4} M	1.19×10^{-4} M	0	0

Supplementary Table 9. Selectivity of the conventional polyamidoxime (H_3IDO) adsorbent at pH 6 in the mixed uranium (VI)-vanadium (V) solution (U(VI), 3.2×10^{-5} M vs. V(V), 5.8×10^{-5} M). The data are taken from Ladshaw et al.[8] The uptake capacity expressed in mol metal per g adsorbent indicate greater selectivity of the H_3IDO adsorbent toward vanadium over uranium.

	Starting Vanadium Concentration	Starting Uranium Concentration	Vanadium Uptake Capacity	Uranium Uptake Capacity
U vs. V	5.8×10^{-5} M	3.2×10^{-5} M	88.162 mg g^{-1} (1.73×10^{-3} mol g^{-1})	183.990 mg g^{-1} (0.77×10^{-3} mol g^{-1})

Supplementary Methods:

Quantum chemical calculations. Electronic structure calculations were performed using Gaussian 09, version D.01[1]. First-principles computations based on density functional theory (DFT) were applied using the B3LYP[2,3] and M06[4] functionals coupled with the Stuttgart small-core (SSC) potentials to account for relativistic effects and the associated contracted [8s/7p/6d/4f] basis sets for uranium, and the 6-311++G(d,p) basis set for other elements. Frequency calculations were performed at the B3LYP/SSC/6-31+G(d) level to ensure that geometries (optimized at the same level) were minima and to compute zero-point energies and thermal corrections to Gibbs free energy, G_{corr}, within the harmonic oscillator approximation using standard formulae based on statistical thermodynamics[5] ($G_{corr} = H_{corr} – TS_{tot},$ where enthalpy ($H_{corr} = E_{tot} + k_BT$) and entropy ($S_{tot}$) include translational, rotational, vibrational, and electronic contributions to the internal thermal energy ($E_{tot} = E_t + E_r + E_v + E_e$) and entropy ($S_{tot} = S_t + S_r + S_v + S_e$), respectively). The obtained values of zero-point energy and thermal corrections to the total energy of species were used to calculate gas-phase free energy change, $\Delta G^o{}_g$, in accord with Supplementary Equation 1:

$$[UO_2(H_2O)_m]^{2+}{}_{(g)} + L^y{}_{(g)} \leftrightarrows [UO_2L(H_2O)_{m-n}]^{2+y}{}_{(g)} + n\ H_2O_{(g)} \qquad (1)$$

where L^y denotes the BHT ligand with a charge of y. Using the optimized gas-phase geometries of the global minimum species, aqueous solvation free energies at 298 K, $\Delta G_{solv}{}^*$, were calculated with the SMD[6] implicit solvation model at B3LYP/SSC/6-31+G(d). The chosen level of theory was confirmed to provide the best overall performance in predicting the stability constant (log β) values for uranium[7-9] complexes with anionic oxygen and amidoxime donor ligands. Complexation free energies in aqueous solution, ΔG_{aq}, and stability constants, log β, were calculated using the methodology described in our previous studies on uranium and vanadium complexes[7-10]. More specifically, this approach utilizes a thermodynamic cycle scheme involving the calculation of the gas-phase free energies and the change in free energy upon transfer of 1 mole of a species from the gas to the aqueous phase under standardized conditions. Once the various free energy terms of the cycle are calculated using quantum chemical methods, then the change in free energy for the aqueous reaction (ΔG_{aq}) can be determined. The log β values are obtained from the relation of log β to ΔG_{aq} by the following equation:

$$\log \beta = -\frac{\Delta G_{aq}}{2.303RT} \qquad (2)$$

Finally, log β^{theor} values in the main text are reported after applying the corresponding regression equations[9] to the calculated log β. These regression equations (1:1 and 1:2 uranyl complexes: [log $\beta^{\mathrm{expt}} = 0.5693\times\log \beta^{\mathrm{calc}}$][9]; 1:2 uranyl complexes carrying an excess negative charge: [log $\beta^{\mathrm{expt}} = 0.6498\times\log \beta^{\mathrm{calc}} - 7.7565$][9]; 1:1 vanadium (V) complexes: [log $\beta^{\mathrm{expt}} = 0.390\times\log \beta^{\mathrm{calc}} - 1.583$][10]) were in turn obtained from the extensive correlations between quantum mechanical calculations of log β and available experimental data.[7,8,10]

Chemical bonding analysis was performed with the natural bond orbital (NBO) method[11,12] at the M06/SSC/6-311++G(d,p) level. NBO analysis provides a good quantitative description of interatomic and intermolecular interactions in accordance with the basic Pauling-Slater-Coulson representations of bond polarization and hybridization.[11,12] The donor-acceptor interaction energy in the NBOs was estimated via second-order perturbation theory analysis of the Fock matrix.[11] For each donor orbital (i) and acceptor orbital (j), the stabilization energy $E^{(2)}$ associated with i→j delocalization is given by:

$$E_{i,j}^{(2)} = -o_i \frac{\langle i | \hat{F}_{(i,j)} | j \rangle^2}{\varepsilon_j - \varepsilon_i} \quad (3)$$

where o_i is the donor orbital occupancy, $\hat{F}_{(i,j)}$ is the Fock operator, and ε_i and ε_j are the orbital energies.

Chemicals. H_2BHT was synthesized as described previously.[13] All organic chemicals and solvents were purchased from commercial sources and were used as received. Uranyl stock solutions were prepared by dissolving UO_3 in perchloric acid or hydrochloric acid, followed by dilution with water. The concentration of uranium was determined by fluorimetry[14] or titration with Arsenazo III[15], and free acid concentration determined by Gran titration. Acid and base stock solutions were standardized before use.

Potentiometric titrations. Potentiometric titrations were performed using an autotitration unit consisting of a double jacket glass titration cell and a Metrohm dosimat (907 Titrando) connected to a pH electrode (Orion model 8102). The temperature of the titration cell was maintained at (25 ± 0.1) °C by circulating water from a constant temperature water bath. An inert atmosphere was maintained in the titration cell by passing Ar gas over the solution to exclude CO_2 during titrations.

Prior to each titration, an acid/base titration with standardized HCl and NaOH was performed to obtain the electrode parameters E^0, γ_H, and γ_{OH}. These parameters allowed the calculation of hydrogen ion concentrations from the measured electromotive force (EMF) in subsequent titrations. In a typical titration, a solution (about 15 mL) containing appropriate amounts of UO_2^{2+} and ligand was titrated with a standardized solution of NaOH. Multiple titrations were conducted with solutions of different concentrations of metal ions. The potentiometric titration data were analyzed to obtain the stability constants of the metal/ligand complexes by the Hyperquad 2008 program.

UV-Visible absorbance spectroscopy. Absorption spectra were acquired on a Cary 50 Spectrophotometer. A 2 mm quartz cuvette was used, and the baseline corrected against a blank solution of water in the cuvette. To prepare the solutions, H_2BHT (3.2 mg, 0.0125 mmol) was suspended in water (15 mL, 0.8 mM), and an aqueous solution of uranyl chloride (0.00625 mmol in 0.1 mL) was added with stirring. The mixture immediately turned an amber color, and upon further stirring the ligand fully dissolved. After 30 minutes, the solution was measured to be pH 5-6. 1 mL aliquots were taken and adjusted to different pH values for absorption spectra. The remainder of the solution at pH 5-6 was allowed to stand for 3 weeks at room temperature, during which large very dark brown blocks suitable for X-ray diffraction formed.

X-ray crystallography. X-ray structural determination was performed on a Bruker APEX diffractometer with a Bruker fixed-Chi 3-Circle goniometer, a Bruker APEX I CCD detector, and a monochromatized fine-focus sealed Mo-kα X-ray source. A crystal was coated with paratone oil and mounted in a Kaptan loop, which was mounted on the goniometer with a nitrogen cryostream held at a temperature of 100 ± 0.5 K. Crystallography data was processed in the WinGX software package, solved using the SHELXTL software package and refined with the SHELXL software package, with semiempirical absorption correction with SADABS, included in SHELXTL.[16] All non-hydrogen atoms were refined anisotropically, and all hydrogen atoms were located in difference maps and refined isotropically.

NMR spectroscopy. ^{1}H NMR spectra in H_2O or H_2O/D_2O mixtures were acquired on a Bruker AV-500 spectrometer (500 MHz) using a WATERGATE pulse sequence for solvent suppression and referenced to an external standard of C_6D_6. ^{1}H NMR spectra in DMSO-d_6 and MeOD were acquired on a Bruker AV-500 spectrometer and referenced to the residual solvent peak. ^{13}C NMR

spectra were acquired on a Bruker DRX-500 spectrometer (126 MHz), referenced to an external standard of C_6D_6. ^{13}C solid state CP MAS NMR spectra were acquired on a Bruker Avance III-400MHz spectrometer (400 MHz).

Synthesis of adsorbent material. The H_2BHT polymeric adsorbent was obtained following the synthetic route depicted in Supplementary Figure 5.

Synthesis of polymer **2**

Polyethylene fibers were irradiated with an electron beam with the combined radiation dose of 40 KGy. The fibers were transferred into the 50 wt% solution of acrylic acid in DMSO. After the reaction the resulting PA-grafted polymer fibers were removed from the solution and were successively washed with acetone, methanol and water. The product was placed into a Soxhlet extractor and was extracted with methanol for additional 72 hours. After drying under high vacuum, the product was obtained as brittle white fibers.

Synthesis of polymer **3**

Polymer **2** (4.1 g) was placed in a 100 mL round bottom flask, CH_2Cl_2 (50 mL) was added followed by a slow addition of 35 mL of $SOCl_2$. The reaction mixture was gently agitated under a gentle flow of argon while maintaining the external temperature at 40 °C. After 24 hours, the reaction mixture was evaporated to dryness under high vacuum affording acylchloride intermediate as light tan brittle fibers.

Synthesis of polymer **4**

Polymer **3** was treated with anhydrous CH_2Cl_2 (150 mL), the reaction mixture was cooled down to 0 C°, followed by the slow addition of N,N-diisopropylethylamine (10 mL) and N-Boc-piperazine (15 g). The reaction mixture was allowed to warm up to room temperature and was gently agitated for additional 12 hours. After that, the solvent was removed and the product was washed with CH_2Cl_2, methanol and vacuum dried. N-Boc protected amine-functionalized fibers **2’** were placed in the 500 mL flask, followed by the addition of anhydrous CH_2Cl_2 (200 mL). Trifluoroacetic acid (20 mL) was slowly added, resulting in gas evolution. After approximately 2 hours the gas evolution ceased. The solvent was decanted and the product was washed with a new

portion of anhydrous CH_2Cl_2. After drying under high vacuum, the product was obtained as brittle tan fibers.

Synthesis of polymer **5**

Polymer **4** (2.0 g) was placed into a 500 mL round bottom flask followed by the addition of anhydrous acetone (300 mL). Reaction mixture was cooled down to -40 °C using dry ice / acetone bath. After that N,N-diisopropylethylamine (8.0 g) was slowly added followed by the addition of 1,3,5-trichlorotriazene (18.4 g). The reaction mixture was allowed to warm up to 0°C. The reaction mixture was gently agitated under a gentle flow of argon while maintaining the external temperature at 0 °C for 24h. the solvent was decanted under argon and the resulting fibers were washed successively with fresh portion of anhydrous acetone, followed by anhydrous CH_2Cl_2. After drying under high vacuum, the product was obtained as brittle tan fibers.

Synthesis of the H_2BHT polymeric adsorbent

Polymeric fibers **5** (1.45 g) were placed into a 200 mL round bottom flask followed by the addition of anhydrous tetrahydrofuran. Reaction mixture was cooled down to 0 °C using water/ ice bath. After that N-methyl hydroxylamine hydrochloride (7.0 g) was added, followed by the slow addition of N,N-diisopropylethylamine (10.34 g). The reaction mixture allowed to warm up to room temperature and was gently agitated under a gentle flow of argon. After completion, the solvent was decanted and the product was successively washed with acetone, methanol, water, acetone and CH_2Cl_2. After drying under high vacuum for 5 hours the product was obtained as light tan brittle fibers.

Uranium adsorption measurements. The starting uranium stock solution was prepared by dissolving 0.2109 g $UO_2(NO_3)_2 \cdot 6H_2O$ in 250 mL deionized water to create a 400 ppm uranium solution. Lower concentration solutions of uranium were obtained by diluting the stock solution with proper amounts of deionized water unless otherwise indicated. The pH levels of all solutions used in sorption isotherm, sorption kinetics, and selectivity studies were adjusted to 6.0 by HNO_3 or NaOH aqueous solution. The concentration of uranium and vanadium for all experiments were detected by inductively coupled plasma-optical emission spectroscopy (ICP-OES). All the adsorption experiments were performed at ambient conditions.

Uranium sorption isotherm. To obtain the adsorption isotherm, 5 mg of adsorbent was placed in 10 mL aqueous solutions of increasing uranium concentrations (1-250 ppm). After the solutions were stirred for 48 hr they were filtered through a 0.45 μm membrane filter and the filtrate was analyzed via ICP-OES to determine the residual uranium concentrations. The amount adsorbed or uptake capacity, q_e (mg g^{-1}), at equilibrium was calculated using Supplementary Equation 4

$$q_e = \frac{(C_0 - C_e) \times V}{m} \quad (4)$$

Where C_0 and C_e are the initial and equilibrium concentrations, respectively, V is the volume of solution used (mL), and m is the mass of adsorbent (g).

Uranium sorption kinetics. 50 mg of adsorbent was added to an Erlenmeyer flask containing 400 mL of a 10 ppm uranium solution. The mixture was then stirred for 48 hr. At increasing time intervals 3 mL aliquots were removed from the mixture, filtered through a 0.45 μm membrane filter, and the filtrate was analyzed by ICP-OES for the remaining uranium concentration.

Recyclability test. Uranium-contacted adsorbent was stirred in 1 M Na_2CO_3 solution (100 mL) overnight. The solid was collected by filtration, washed with deionized water, and dried under vacuum for further use. Subsequent adsorption experiments were performed after base treatment and contacted with a solution of ~10 ppm (400 mL) uranium. The recyclability test was repeated 3 times with the final result (3rd recycling) reported in Supplementary Table 7.

Competitive adsorption (selectivity) test. A mixed metal solution was made with $UO_2(NO_3)_2 \cdot 6H_2O$ and Na_3VO_4 with equal concentrations of 10 ppm. 6.5 mg of adsorbent was added to 200 mL of the mixed metal solution, this was then stirred overnight. The solutions were filtered through a 0.45 μm membrane filter, and the filtrate was analyzed by ICP-OES for the remaining uranium and vanadium concentrations. The removal efficiency was calculated by Supplementary Equation 5

$$\text{Removal efficiency (\%)} = \frac{C_0 - C_e}{C_0} \times 100 \quad (5)$$

Where C_0 and C_e are the initial and equilibrium concentrations, respectively.

Additional competitive adsorption experiments were performed to confirm the observed selectivity in the presence of high concentration of vanadium (V) species in solution. A solution was made

by mixing $UO_2(NO_3)_2 \cdot 6H_2O$ and Na_3VO_4 at [V]/[U] ~ 3/1 concentration ratio ([V] = 1.18×10^{-4} M; [U] = 0.42×10^{-4} M). 50 mg of adsorbent was added to 400 mL of the mixed metal solution, this was then stirred overnight. The solutions were filtered through a 0.45 μm membrane filter, and the filtrate was analyzed by ICP for the remaining vanadium concentrations (reported in Supplementary Table 8).

Supplementary Note 1. Although there is no experimental evidence for the displacement of the V=O oxido bonds by H_2BHT, we considered the possible formation of a hypothetical 1:2 non-oxido $V(BHT)_2^+$ complex to provide a complete picture of the V(V) complexation by the H_2BHT ligand. First, we applied our computational protocol[10] to predict log β^{theor} values of the conventional 1:1 dioxovanadium complexes. The obtained results (log β^{theor} [$VO_2(BHT)^-$] = 17.3; log β^{theor} [VOOH(BHT)] = 19.1 with respect to $H_2VO_4^-$ species) are in good agreement with the experimental data reported relative to $H_2VO_4^-$ species by Nikolakis et al.[13] (log β^{expt} [$VO_2(BHT)^-$] = 17.87 (1); log β^{expt} [VOOH(BHT)] = 19.39 (6). The corresponding log β^{theor} for the non-oxido $V(BHT)_2^+$ complex can be obtained by a combination of the reactions for which ΔG_{aq} is experimentally known or was assessed using DFT calculations. The complexation free energy, ΔG_{aq8}, and subsequently log β^{theor} for the formation of the $V(BHT)_2^+$ complex was found by combination of the following reactions ($\Delta G_{aq9} = 2\Delta G_{aq6} + 2G_{aq7} + \Delta G_{aq8}$):

ΔG_{aq6} : $H_2VO_4^- + 2H^+ \leftrightarrows VO_2^+ + 2H_2O$ (6)

ΔG_{aq7} : $VO_2^+ + HBHT^- \leftrightarrows VOOH(BHT)$ (7)

ΔG_{aq8} : $VOOH(BHT) + VOOH(BHT) \leftrightarrows V(BHT)_2^+ + H_2VO_4^-$ (8)

ΔG_{aq9} ($\Delta G_{aq9} = 2\Delta G_{aq6} + 2G_{aq7} + \Delta G_{aq8}$):

$\mathbf{H_2VO_4^- + 4H^+ + 2HBHT^- \rightleftharpoons V(BHT)_2^+ + 4H_2O}$ (9)

where ΔG_{aq6} is experimentally known value from Supplementary Table 5 (at 25 °C and I = 0 M), ΔG_{aq7} was determined using our methodology from supplementary reference 10, and the free energy, ΔG_{aq8}, was calculated at the M06/SSC/6-311++G(d,p) level of theory. The obtained ΔG_{aq8} value of +13.33 kcal mol^{-1} indicate a thermodynamically unfavorable process. This is in direct contrast to the H_3IDO ligand, for which ΔG_{aq} of a similar reaction ($VOOH(HIDO)^- + VOOH(HIDO)^- \leftrightarrows V(IDO)_2^- + H_2VO_4^-$) was calculated to be -0.11 kcal mol^{-1} at the same level of theory. In addition, the generated species distribution diagrams (Supplementary Fig.2), constructed by incorporating log β^{theor} of the V(V)/H_2BHT complexes (Supplementary Table 5) along with the experimental hydrolysis constants for mononuclear vanadium(V) species, show that only 1:1 vanadium complexes are the dominant species at both [H_2BHT]/[V] = 1/1 and [H_2BHT]/[V] = 100/1 concentration ratios over the pH range of ~3–10. It is also worth noting that the speciation

data suggest the feasibility of removing all vanadium from the H_2BHT fiber by increasing pH of the solution, which contrasts with the H_3IDO-functionalized adsorbent materials.

Supplementary Note 2. In order to corroborate changes in pH and investigate the electronic structure of the U(VI)-H_2BHT complexes, we investigated their UV-Visible absorption spectra. During the titrations, a deep amber color was observed, and we sought to investigate if it varied at different acidities to use as another characterization of solution binding. Spectrophotometric titrations can be used in a similar manner as potentiometric titrations, by titrating acid into a mixture and fitting it to calculated or known absorption spectra of the individual components. It should be noted that we tend to use the spectrophotometric data to supplement those from potentiometry, because it is not always possible to differentiate species that differ only in the number of protons. Besides, the sensitivity of spectrophotometry may be low if the absorptivity of species, e.g., those of uranyl whose color tend to be weak and vary less than other actinide or transition metal species. [17-19]

The free H_2BHT ligand is colorless and has no visible absorption but absorbs strongly in the ultraviolet region due to its conjugated aromatic core (Supplementary Fig. 4). Uranyl also has weak visible absorption, appearing pale yellow in solution. In contrast, when they are mixed, darker amber colors are observed. The dramatic change and increase in intensity of color is the result of new ligand-metal charge transfer transitions that can occur. Interestingly, the UV absorption of the free ligand extends further towards the visible region than in complexes, with an absorption cutoff at about 320 nm, as opposed to 290-300 nm when uranyl is present. This indicates that the transitions observed in the triazine core are affected by the presence of uranyl, either through participation of the nitrogen atom in bonding or distortions in geometry upon binding which affects conjugation throughout the molecule.

Although full spectrophotometric titrations were not performed, the spectra can nonetheless corroborate the different stoichiometries observed over a wide pH range. As the pH is raised from acidic to neutral, the major absorption band at 450 nm appears. The absorption does not change significantly with protonation state as the pH changes, however, the absorption decreases as the pH is increased further, and the band shifts to 425 nm in more basic solution, as the 1:2 uranyl:H_2BHT complexes are formed. This absorption is slightly weaker, which is consistent with previously observed symmetric vs. asymmetric actinyl species.[17,18] Several absorption bands of

lower intensity can be seen around 340 nm and 640 nm, the former of which contributes to the overall yellow-amber color observed.

Supplementary Note 3. For the competitive (U(VI) vs. V(V)) adsorption test 6.5 mg of the H_2BHT adsorbent was added to 200 mL of the mixed metal solution of equal concentrations (10 ppm) of $UO_2(NO_3)_2{\cdot}6H_2O$ and Na_3VO_4). The experiments at pH ~6 were done two times, both showing a significant drop in uranium concentration from 10 ppm to ~0.15 ppm (see Supplementary Table 7). The vanadium (V) concentration remained unchanged (Supplementary Table 8), indicating no adsorption of vanadium ions by the H_2BHT polymer. This behavior can likely be explained by the difference in the formation constant values for uranium *vs.* vanadium complexes with the H_2BHT functional group. According to our theoretical and experimental studies on small molecule analogs, vanadium binding constant for H_2BHT ($\log\beta$ = 17.9)[13] is much lower than the uranyl binding strength with two H_2BHT ligands ($\log\beta$ = 41.9, Table 1 in the main text). Therefore, vanadium would not likely compete with uranium for H_2BHT active sites, explaining impressive selectivity of the developed H_2BHT-functionalized polymer toward uranyl over vanadium species in aqueous medium.

The adsorbent was fully regenerated after washing procedure (1M Na_2CO_3), achieving a similar uranium uptake capacity even after the 3rd recycling for 10 ppm uranyl solution (Supplementary Table 7 and Fig. 3e in the main text).

Supplementary References

1. Frisch, M. J. et al. Gaussian 09 Revision D.01, Wallingford, CT, Gaussian, Inc., 2009.
2. Becke, A. D. Density-functional Thermochemistry. III. The Role of Exact Exchange. *J. Chem. Phys.* **98**, 5648–5652 (1993).
3. Lee, C., Yang, W. & Parr, R. G. Development of the Colle-Salvetti Correlation-energy Formula into a Functional of the Electron Density. *Phys. Rev. B* **37**, 785–789 (1988).
4. Zhao, Y. & Truhlar, D. G. The M06 suite of density functionals for main group thermochemistry, thermochemical kinetics, noncovalent interactions, excited states, and transition elements: two new functionals and systematic testing of four M06-class functionals and 12 other functionals. *Theor. Chem. Acc.* **120**, 215–241 (2008).

5. McQuarrie, D. A. & Simon, J. D. Molecular Thermodynamics, University Science Books, USA, 1999.

6. Marenich, A. V., Cramer, C. J. & Truhlar, D. G. Universal Solvation Model Based on Solute Electron Density and a Continuum Model of the Solvent Defined by the Bulk Dielectric Constant and Atomic Surface Tensions. *J. Phys. Chem. B* **113**, 6378–6396 (2009).

7. Vukovic, S., Hay, B.P. & Bryantsev, V.S. Predicting Stability Constants for Uranyl Complexes Using Density Functional Theory. *Inorg. Chem.* **54**, 3995–4001 (2015).

8. Mehio, N. et al. Quantifying the Binding Strength of Salicylaldoxime-Uranyl Complexes Relative to Competing Salicylaldoxime-Transition Metal Ion Complexes in Aqueous Solution: A Combined Experimental and Computational Study. *Dalton Trans.* **45**, 9051–9064 (2016).

9. Ladshaw, A. P. et al. First-Principles Integrated Adsorption Modeling for Selective Capture of Uranium from Seawater by Polyamidoxime Sorbent Materials. *ACS Apl. Mat. & Inter*. **10**, 12580–12593 (2018).

10. Ivanov, A.S. & Bryantsev, V.S. Assessing Ligand Selectivity for Uranium over Vanadium Ions to aid in the Discovery of Superior Adsorbents for Extraction of UO_2^{2+} from Seawater. *Dalton Trans.* **45**, 10744–10751 (2016).

11. Reed, A. E., Curtiss, L. A. & Weinhold, F. Intermolecular Interactions from a Natural Bond Orbital, Donor-Acceptor Viewpoint. *Chem. Rev.* **88**, 899–926 (1988).

12. Foster, J. P. & Weinhold, F. Natural Hybrid Orbitals. *J. Am. Chem. Soc.* **102**, 7211–7218 (1980).

13. Nikolakis, V. A. et al. Vanadium (V) Compounds with the Bis-(hydroxylamino)-1, 3, 5-triazine Ligand, H_2bihyat: Synthetic, Structural, and Physical Studies of [V_2VO_3(bihyat)$_2$] and of the Enhanced Hydrolytic Stability Species cis-[V^VO_2 (bihyat)]$^-$. *Inorg. Chem.* **47**, 11698–11710 (2008).

14. Smith, N. A., Cerefice, G. S. & Czerwinski, K. R. *J. Radioanal. Nucl. Chem.* **295**, 155, 1560 (2013).

15. Savvin, S. Analytical Use of Arsenazo III: Determination of Thorium, Zirconium, Uranium and Rare Earth Elements. *Talanta* 8, 673–685 (1961).

16. Sheldrick, G. M. SHELXT–Integrated Space-Group and Crystal-Structure Determination. *Acta Crystallogr. Sect. A Found. Crystallogr.* **71**, 3–8 (2015).

17. Xu, C., Tian, G., Teat, S. J. & Rao, L. Complexation of U (VI) with Dipicolinic Acid: Thermodynamics and Coordination Modes. *Inorg. Chem.* **52**, 2750–2756 (2013).

18. Endrizzi, F. & Rao, L. Chemical Speciation of Uranium (VI) in Marine Environments: Complexation of Calcium and Magnesium Ions with $[(UO_2)(CO_3)_3]^{4-}$ and the Effect on the Extraction of Uranium from Seawater. *Chem. - A Eur. J.* **20**, 14499–14506 (2014).

19. Sun, X. et al. Quantifying the Binding Strength of U (VI) with Phthalimidedioxime in Comparison with Glutarimidedioxime. *Dalton Trans.* **43**, 551–557 (2014).
